# Population Structure and Phylogeography in Nassau Grouper (*Epinephelus striatus*), a Mass-Aggregating Marine Fish

**DOI:** 10.1371/journal.pone.0097508

**Published:** 2014-05-15

**Authors:** Alexis M. Jackson, Brice X. Semmens, Yvonne Sadovy de Mitcheson, Richard S. Nemeth, Scott A. Heppell, Phillippe G. Bush, Alfonso Aguilar-Perera, John A. B. Claydon, Marta C. Calosso, Kathleen S. Sealey, Michelle T. Schärer, Giacomo Bernardi

**Affiliations:** 1 Department of Ecology and Evolutionary Biology, University of California Santa Cruz, Santa Cruz, California, United States of America; 2 Scripps Institution of Oceanography, University of California San Diego, La Jolla, California, United States of America; 3 School of Biological Sciences, University of Hong Kong, Hong Kong, China; 4 Department of Zoology/Marine Biology, University of the Virgin Islands, St. Thomas, Virgin Islands, United States of America; 5 Department of Fisheries and Wildlife, Oregon State University, Corvallis, Oregon, United States of America; 6 Protection and Conservation Unit, Cayman Islands Department of the Environment, Grand Cayman, British West Indies; 7 Departmento de Biología Marina, Universidad Autónoma de Yucatán, Mérida, Yucatán, México; 8 The School for Field Studies, Center for Marine Resource Studies, South Caicos, Turks and Caicos Islands; 9 Department of Biology, University of Miami, Coral Gables, Florida, United States of America; 10 Puerto Sea Grant, University of Puerto Rico, Mayagüez, Puerto Rico; Temasek Life Sciences Laboratory, Singapore

## Abstract

To address patterns of genetic connectivity in a mass-aggregating marine fish, we analyzed genetic variation in mitochondrial DNA (mtDNA), microsatellites, and single nucleotide polymorphisms (SNPs) for Nassau grouper (*Epinephelus striatus*). We expected Nassau grouper to exhibit genetic differentiation among its subpopulations due to its reproductive behavior and retentive oceanographic conditions experienced across the Caribbean basin. All samples were genotyped for two mitochondrial markers and 9 microsatellite loci, and a subset of samples were genotyped for 4,234 SNPs. We found evidence of genetic differentiation in a Caribbean-wide study of this mass-aggregating marine fish using mtDNA (F_ST_ = 0.206, *p*<0.001), microsatellites (F_ST_ = 0.002, *p* = 0.004) and SNPs (F_ST_ = 0.002, *p* = 0.014), and identified three potential barriers to larval dispersal. Genetically isolated regions identified in our work mirror those seen for other invertebrate and fish species in the Caribbean basin. Oceanographic regimes in the Caribbean may largely explain patterns of genetic differentiation among Nassau grouper subpopulations. Regional patterns observed warrant standardization of fisheries management and conservation initiatives among countries within genetically isolated regions.

## Introduction

Effective management of marine populations requires knowledge of the extent of connectivity among locations [1], [2]. While connectivity is extremely difficult to directly estimate in marine populations, molecular markers and associated analytical techniques provide indirect estimates of larval movement and dispersal of organisms [3], [4]. A combination of biotic and abiotic factors likely contribute to patterns of connectivity observed in marine systems. Ocean currents [5], [6], larval behavior [7], pelagic larval duration (PLD) [8], isolation by distance [9], [10] and historical vicariance [11], [12] in particular may play important roles in either enhancing long distance dispersal or limiting exchange among populations.

Group spawning behavior exhibited in some families of reef fish may further restrict connectivity between localities. A spawning aggregation is a gathering of conspecific fish for the purposes of reproduction [13]. Such aggregations are ephemeral and can be highly synchronized and restricted in space and time [13], [14]. Adult fish migrate to spawning sites such that a spawning aggregation is typically an amalgamation of all reproductive individuals in a given geographic area (i.e. catchment area *sensu* Nemeth [15]). Thus, larvae produced from a given (sub)population are concentrated at spawning sites, with ocean currents, PLD, and larval behavior potentially influencing dispersal patterns of larvae spawned at an aggregation site. If the aforementioned factors facilitate isolation between adjacent catchment areas, there is increased potential for genetic subdivision among subpopulations and decreased likelihood that settling larvae originate from other locations [16].

The broad geographic distribution of Nassau grouper (*Epinephelus striatus*) throughout the Caribbean Sea and western Atlantic Ocean makes it a suitable model species to investigate genetic subdivision in a mass-aggregating species. Nassau grouper typically aggregate to spawn for about one week per month over a period that lasts up to three months, in association with water temperature, the moon phase and maximal tidal amplitudes [17], [18]. Individuals can migrate long distances to spawn (up to 220 km [19]) and larvae remain in the water column for 35 to 40 days before settling [20]. Additionally, knowledge of genetic subdivision is particularly important as results can contribute to fisheries management of a commercially exploited species. Historically, Nassau grouper spawning aggregations may have consisted of up to tens of thousands of individuals [17], [21], however targeted fishing of spawning aggregations has drastically decreased population sizes and extirpated one third of all known aggregations [22]. As a result of its decline, the Nassau grouper is now listed as Endangered on the IUCN Red List. Overfishing of such an important top predator has already impacted reef fish community structure [23], [24], census population sizes, and may negatively impact levels of genetic diversity [25], long-term viability and the economic and food benefits of this once common species.

To address patterns of connectivity in a mass-aggregating marine fish we analyzed patterns of genetic variation in mitochondrial DNA (mtDNA), microsatellites, and single nucleotide polymorphisms (SNPs) for Nassau grouper. Limited genetic work on Nassau grouper has focused on a narrow subset of the species’ geographic range and used only a few microsatellite loci. These previous studies have failed to resolve any regional or local scale genetic differentiation between subpopulations [26]. In our study, we dramatically increased both the geographic distribution of samples and the number of molecular markers analyzed to determine whether Nassau grouper subpopulations represented in spawning aggregations are genetically differentiated. Our expectation was that Nassau grouper would exhibit both local and regional differentiation among subpopulations due to its spatially and temporally restricted reproductive behavior, as well as the variety of oceanographic conditions experienced across the broad Caribbean basin (approximately 2.75 million km^2^). Given the decline of Nassau grouper across the region, our findings will have major implications for designing spatially explicit management and conservation strategies. While there are few obvious physical barriers to long-distance dispersal between most aggregation sites, if substantial genetic structure is observed among aggregations then protection of aggregation sites may be the only means by which 1) distinct subpopulations can be maintained and 2) local natural resource management authorities can effectively ensure the long-term sustainability of fisheries dependent on aggregating species.

## Materials and Methods

### Sample Collection and DNA Extraction

**Figure 1:**
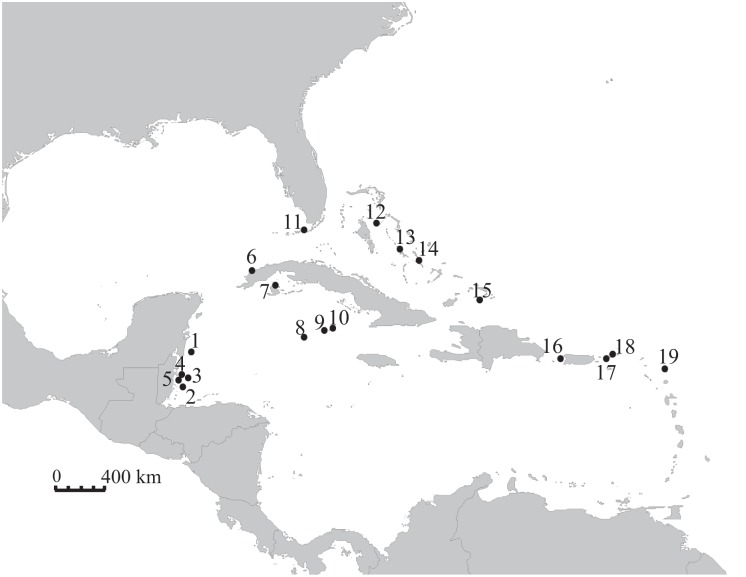
Nassau grouper sampling localities in the Caribbean Sea. Sampling localities include: 1) Chinchorro Bank, Mexico, 2) Glover’s Reef, Belize, 3) Lighthouse Reef, Belize, 4) Turneffe Atoll, Belize, 5) Caye Glory, Belize, 6) Corona San Carlos, Cuba, 7) Pardon del Medio, Cuba, 8) Grand Cayman, 9) Little Cayman, 10) Cayman Brac, 11) Florida Keys, 12) Dog Rocks, N. Exuma, 13) Lee Stocking, Bahamas, 14) Long Island, Bahamas, 15) South Caicos, 16) Bajo de Sico, Puerto Rico, 17) Grammanik Bank, U.S. Virgin Islands, 18) N. of St. Thomas, British Virgin Islands and 19) Antigua.

All sampling protocols for this scientific study were approved by IACUC at the University of California Santa Cruz. We acquired a total of 620 Nassau grouper tissue samples (fin clips or muscle) from 19 sites across 9 countries, with samples collected between 1993 and 2013 (Fig. 1, Table 1). Collections were conducted with permits from the Cayman Islands Conservation Board, the Bahamas Department of Marine Resources, the Turks and Caicos Department of Environment and Coastal Resources, and the National Marine Fisheries Service. Specific permissions were not required to collect at certain sampling sites (Sites 2–7,11, 13, 14, 18, 19) as either samples were acquired before a time that collection permits were required or samples were acquired during fishery-dependent activities. Tissue samples were either directly collected from spawning aggregations or in the time immediately before or after the fishery closure, depending on the year they were collected and the local fisheries management in place. We obtained samples from hook and line fisheries, from fish that were caught and released in the pursuit of scientific study, from Antillean fish traps, or while using closed-circuit rebreathers. Samples were stored in a sarcosyl-urea solution, dimethyl sulfoxide (DMSO) solution or 95% ethanol. Sarcosyl-urea and DMSO samples were stored at room temperature. Samples in 95% ethanol were stored at −20°C. Genomic DNA was isolated following the manufacturer’s protocol for the Qiagen DNeasy blood and tissue kit.

**Table 1:** Sampling localities for Nassau grouper.

Region	Sampling Site	Sampling Year	N_mtDNA_	N_msat_	N_SNPs_
MesoamericanReef	1. Chinchorro Bank, Mexico*	2013	7	24	0
	2. Glover’s Reef, Belize	1994	21	59	31
	3. Lighthouse Reef, Belize	1993	4	32	0
	4. Turneffe Atoll, Belize	1993	5	29	0
	5. Caye Glory, Belize	1995	12	26	0
CentralCaribbean	6. Corona SanCarlos, Cuba	1996	14	24	0
	7. Pardon delMedio, Cuba	1996	17	41	0
	8. Grand Cayman, Cayman Is.	2008	8	9	0
	9. Little Cayman, Cayman Is.	2005	72	61	14
	10. Cayman Brac, Cayman Is.	2008	27	28	0
	11. Florida Keys, U.S.A.	1994	31	38	0
Bahamas	12. Dog Rocks,Northern Exuma	2011	4	19	0
	13. Lee Stocking	1994	4	23	0
	14. Long Island	2000	23	37	32
EasternCaribbean	15. South Caicos,Turks and Caicos^+^	2011	32	50	0
	16. Bajo de Sico, Puerto Rico^+^	2013	10	10	0
	17. Grammanik Bank,U.S.Virgin Is.^+^	2010	72	58	0
	18. N. of St. Thomas,British Virgin Is.	1999	0	8	0
	19. Antigua^+^	2013	32	44	31

Sample sizes utilized for mitochondrial DNA (N_mtDNA_), microsatellites (N_msat_) and SNP (N_SNPs_) analyses. Majority of tissue samples were stored in sarcosyl-urea unless denoted with (*) for dimethyl sulfoxide (DMSO) or (^+^) for 95% ethanol.

### Genotyping and Data Analysis for Mitochondrial Markers

We genotyped samples for two mitochondrial markers: ATPase and cytochrome *b*. We amplified a 634 bp fragment of ATPase using primers L8331 and H9236 [27]. Polymerase chain reaction (PCR) used the following thermocycler parameters: an initial hold at 94°C/5 min, 35 cycles of 94°C/30 sec, 54°C/30 sec, 72°C/30 sec, followed by a final extension of 72°C/7 min. We then amplified a 785 bp fragment of cytochrome *b* using primers Gludgl and CB3H [28]. Thermocycler parameters were as follows: initial hold at 94°C/5 min, 35 cycles of 94°C/45 sec, 45°C/45 sec, 72°C/45 sec, followed by a final extension of 72°C/7 min. Successfully amplified PCR products were sequenced on an ABI 3730xl DNA analyzer at the UC Berkeley DNA Sequencing Facility. Sequences were proofread and aligned, using the software Geneious (version 5.6, Biomatters Ltd.). We used jModeltest 0.1.1 [29] to select the nucleotide substitution model that best fit the ATPase and cytochrome *b* datasets. ATPase and cytochrome *b* sequences were ultimately analyzed as concatenated sequences, for a combined total of 1,419 bp, because patterns of genetic variation observed in both markers were best explained by the same nucleotide substitution model.

We calculated molecular diversity indices including haplotype diversity (h) and nucleotide diversity (π) using Arlequin [30]. We corrected haplotype diversity (h*) using a rarefaction approach, as implemented in CONTRIB [31], to account for differences in sample size between sites based on a minimum sample size of *n = *8 per site. We then assessed phylogenetic relationships among sequences by generating a haplotype network using the software packages pegas [32] and geiger [33] in R.

### Genotyping and Data Analysis for Microsatellite Loci

All samples were genotyped for nine polymorphic microsatellites previously designed for Gulf coney (*Hyporthodus acanthistius*), following published PCR protocols [34]. Amplification products were sized on an ABI 3730xl DNA analyzer at the UC Berkeley DNA Sequencing Facility using the size standard LIZ-500 (Applied Biosystems). Microsatellites were scored using GeneMapper version 3.7 (Applied Biosystems) and tested for null alleles, large allele dropout and scoring errors using Micro-Checker [35]. We calculated number of alleles, expected heterozygosity (H_e_), observed heterozygosity (H_O_) and performed exact tests to detect deviations from the expectations of Hardy-Weinberg equilibrium (HWE) using Arlequin [30].

### Genotyping and Data Analysis for Single Nucleotide Polymorphisms (SNPs)

Restriction site associated DNA (RAD) tag libraries were created using the protocol described in Hohenlohe et al. [36]. Genomic DNA was collected from a subset of tissues collected (n = 108) from four localities: Little Cayman (site 9), Glover’s Reef, Belize (site 2), Long Island, Bahamas (site 14) and Antigua (site 19). DNA from each individual was digested with the restriction enzyme *SbfI*, and fragments were ligated to a unique, 6 bp barcoded adapter. The pooled single end libraries were sequenced on an Illumina Genome Analyzer IIx.

SNP discovery and genotyping were performed using modified Perl scripts (described in Miller et al. [37]) and using the software package Stacks [38]. All sequenced fragments were first trimmed from the 3′ end to a length of 92-bp. Low quality reads with a probability of sequencing error greater than 0.10% (Phred score = 33) were then filtered out. Reads without an exact match to the 6-bp barcode and 6-bp *SbfI* restriction site were also filtered out. For all remaining fragments, the combined 12-bp sequence (barcode plus restriction site) was then removed. Final filtered reads (80 bp) were then utilized in a population genomic analysis executed in Stacks. Putative SNPs were selected that met the following criteria: minimum depth coverage of 6X, present in at least 80% of individuals and present in individuals from all four sampling localities.

### Population Structure

We estimated global and pairwise estimates of F_ST_ for all three marker types. Statistical significance of pairwise F_ST_ values was assessed after Bonferroni correction (mtDNA and microsatellites, critical *p = *0.00029; SNPs, critical *p* = 0.00833). To test for evidence of regional genetic structure, we implemented an analysis of molecular variance (AMOVA) in Arlequin. We tested a four-region hypothesis based on genetically isolated regions identified in previous genetic studies in the Caribbean Sea [39]–[41]. Sampling localities were grouped into the following regions: Mesoamerican Reef [sites 1–5], central Caribbean [sites 6–11], the Bahamas [sites 14–16] and eastern Caribbean [sites 12, 13, 17–19]. We used two additional methodologies to determine patterns of genetic differentiation among sites without *a priori* geographic assumptions about regional groups. Both methods utilized both the mtDNA and microsatellite datasets. First we used a computational geometry approach implemented in the software package Barrier [42]. Delaunay triangulation and Voronoi tessellation were used to visualize patterns of geographic variation. Triangular pairwise geographic distance matrices were generated using a Geographic Distance Matrix Generator [43] and pairwise genetic distance matrices were generated in Arlequin. Datasets were bootstrapped and 1,000 bootstrapped genetic distance matrices were utilized. Ranking and strength of observed barriers was determined based on methods described in Manni et al. [42]. Next, we used a simulated annealing approach to maximize among group variance, as implemented by the software SAMOVA [44]. Inferred groups are then tested for significance *a posteriori* via AMOVA.

We performed partial mantel tests to determine whether significant isolation by distance exists among localities for all three marker types. Because hierarchical population structure can introduce bias to isolation by distance analyses [45], partial mantel tests assess the correlation between geographic distance and genetic distance while also controlling for the effect of hierarchical population structure. Tests were implemented using the vegan package in R [46]. Pairwise genetic distances were estimated in Arlequin and geographic distances between sampling localities were calculated using a Geographic Distance Matrix Generator.

## Results

### Mitochondrial DNA

**Figure 2:**
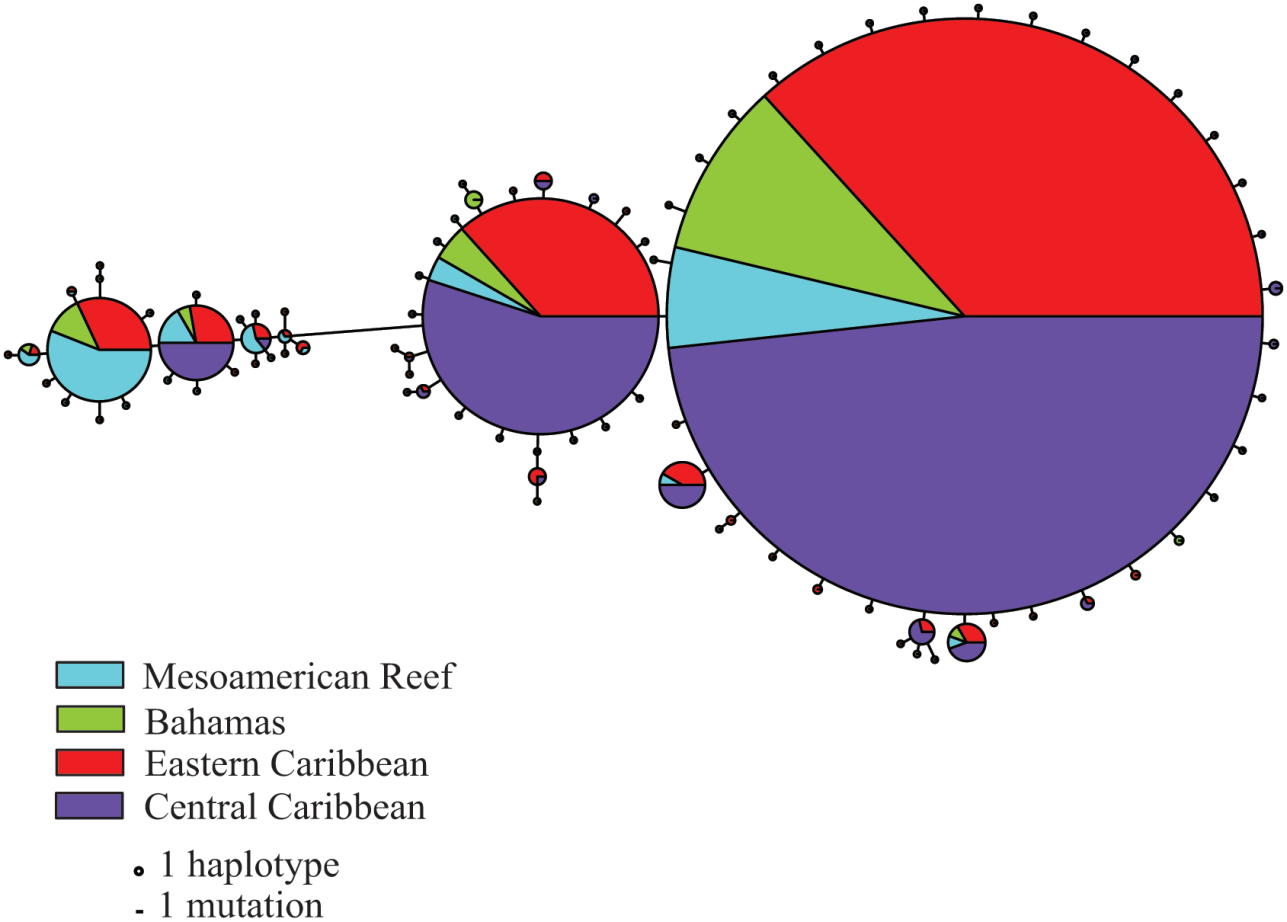
Haplotype network for Nassau grouper. Circles are sized proportionally to the number of individuals that possess each haplotype. The pie chart within each haplotype represents the relative frequency of individuals from each color-coded region. A scale is provided to determine the number of mutations separating each haplotype.

We sequenced a combined total of 1,419 bp for ATPase and cytochrome *b* in 395 individuals (Genbank KF706690–KF707475), resulting in a total of 89 haplotypes (Table 2, Fig. 2). The average distance observed between haplotypes was 1 to 2 bp, with a maximum distance of 16 bp. The two most abundant haplotypes were observed in all sampling localities. There was also a noticeable shift in the proportion of individuals associated with the Mesoamerican Reef for a given haplotype (Fig. 2 in blue), in particular across a 16-bp break. The number of haplotypes (nH), corrected haplotype diversity (h*) and nucleotide diversity (π) are reported in Table 2. Nucleotide diversity ranged from 0.0005 (sites 3 and 8) to 0.0089 (site 17) and showed a decreasing east to west longitudinal trend across the Caribbean basin (R^2^ = 0.271, *p* = 0.016). Corrected haplotype diversity ranged from to 0.500 (site 13) to 0.954 (site 19).

**Table 2:** Molecular diversity indices for concatenated mitochondrial markers for Nassau grouper.

Sampling Site	*n*	nH	h*	π
1. Chinchorro Bank	7	5	0.857	0.0071±0.004
2. Glover’s Reef	21	9	0.852	0.0070±0.004
3. Lighthouse Reef	4	2	0.667	0.0005±0.001
4. Turneffe Atoll	5	3	0.700	0.0006±0.001
5. Caye Glory	12	6	0.758	0.0008±0.001
6. Corona San Carlos	14	6	0.780	0.0009±0.001
7. Pardon del Medio	17	6	0.721	0.0032±0.002
8. Grand Cayman	8	6	0.893	0.0005±0.001
9. Little Cayman	72	26	0.776	0.0011±0.001
10. Cayman Brac	27	12	0.732	0.0008±0.001
11. Florida Keys	31	12	0.817	0.0011±0.001
12. Dog Rocks	4	3	0.833	0.0088±0.006
13. Lee Stocking	4	2	0.500	0.0035±0.001
14. Long Island	23	11	0.834	0.0052±0.003
15. South Caicos	32	14	0.823	0.0053±0.003
16. Bajo de Sico	10	6	0.844	0.0064±0.003
17. Grammanik Bank	72	20	0.737	0.0089±0.001
18. N. of St. Thomas	-	-	-	-
19. Antigua	32	22	0.954	0.0070±0.003

Sample location, number of specimens (*n*), number of haplotypes (nH), corrected haplotype diversity (h*) and nucleotide diversity (π), as reported by Arlequin 3.5.

### Microsatellites

All nine microsatellite loci were polymorphic in Nassau grouper. Microsatellite sequences were deposited to Genbank (FJ178389, FJ178390, JX041258–60, JX041262, JX041282, FJ711588, FJ711590). The total number of alleles per locus per site ranged from 4 to 25 (Table S1 in File S1). Allelic richness per locus ranged from 7.9 (site 8) to 16.2 (site 16) and there appeared to be no geographic trend in values. Observed heterozygosities ranged from 0.11 (site 8) to 1.00 (sites 3, 8, 12, 16). No significant linkage disequilibrium was observed between loci within subpopulations (*p*>0.05, after Bonferroni correction). There was also no evidence of scoring error or null alleles. There was also no evidence of scoring error or null alleles. Significant departures from HWE were observed for 15 out of 171 exact tests (p<0.05). One locus (A108) departed from HWE in 6 of 19 populations, with other loci departing from HWE in four or less populations.

### Single Nucleotide Polymorphisms

RAD tag libraries were created by individually barcoding 108 individuals from 4 spawning sites. One lane of sequencing yielded more than 221 million reads. Raw data will be made available upon request. The raw dataset is not currently publicly accessible, as it is still being utilized in additional analyses beyond the scope of this project. Stringent quality filtering of the raw dataset left a remaining 9.1 million reads. Within each population, we identified an average of 58,305±4,594 stacks, where each stack is comprised of filtered reads representing a potential locus. After specifying a depth coverage of no less than 6X and SNP presence in at least 80% of all individuals using the populations script in Stacks, we identified a total of 4,234 SNPs within the RAD tag sequences. All identified SNPs were variable among individuals from all four localities.

### Population Structure

We detected genetic differentiation between subpopulations using mtDNA (F_ST_ = 0.206, *p*<0.001), microsatellites (F_ST_ = 0.002, *p* = 0.004) and SNPs (F_ST_ = 0.002, *p* = 0.014) (Table 3). Pairwise Φ_ST_ and F_­ST_ comparisons confirmed patterns observed in global estimates from the mtDNA and SNP datasets (Table S2 and S3 in File S1). After Bonferroni correction, only 52 of 330 total pairwise comparisons were significant (47, 0 and 5 significant comparisons for mtDNA, microsatellites, and SNPs, respectively). The majority of significant pairwise comparisons represent between-region comparisons (e.g. between spawning aggregations in the Mesoamerican Reef and eastern Caribbean). A closer examination revealed that Caye Glory, Belize was highly divergent, with 12 out of 18 statistically significant pairwise comparisons. We found no evidence for isolation by distance using partial mantel tests in the mtDNA (r = −0.04536, *p* = 0.670), microsatellite (r = 0.023, *p* = 0.990) or SNP datasets (r = 0.095, *p* = 0.790).

**Table 3:** AMOVA results for mitochondrial DNA, microsatellites and SNPs.

	d.f.	var	var%	F_ST_	*P*-value
*mtDNA*					
Among populations	15	0.8216	20.29	0.2060	<0.0001*
Within populations	377	2.2612	79.40	0.2036	<0.0001*
Total	394	3.9770			
*Microsatellites*					
Among populations	16	0.0078	0.20	0.0023	0.0039*
Within populations	1221	3.8413	99.77	0.0020	0.0088*
Total	1239	3.8502			
*SNPs*					
Among populations	2	0.1159	0.02	0.0020	0.0140*
Within populations	212	159.9480	1.05	-0.0490	1.0000
Total	215	157.4285			

Degrees of freedom (d.f.), variance components (var), percent variation (var %) and *F*-statistics to test for evidence of genetic differentiation among Nassau grouper subpopulations using mitochondrial DNA, microsatellites and SNPs. (*) denotes statistical significance of *p<*0.05.

We tested for evidence of regional genetic differentiation using AMOVAs (Table 4). The four-region model, with genetic differentiation of the Mesoamerican Reef, central Caribbean, eastern Caribbean and a distinct Bahamas enclave, was supported by both the mtDNA and microsatellite datasets (*p*<0.05). Regional differences accounted for approximately 18.09% of the variance in mtDNA and 0.10% of the variance in microsatellite datasets. Approximately 23.09% of the variance in mtDNA and 0.15% of the variance in the microsatellite datasets could also be explained by variance among samples within groups.

**Table 4:** AMOVA results to test for regional patterns of genetic differentiation.

*mtDNA*	Source of variation	d.f.	var%	*P*-value
MesoAmerican Reef,Central Caribbean,	Among groups	3	18.09	0.04208*
Bahamas,Eastern Caribbean	Among populationswithin groups	14	23.19	<0.00001*
	Within populations	377	58.72	<0.00001*
***Microsatellites***	**Source of variation**	**d.f.**	**var%**	***P*** **-value**
MesoAmerican Reef,Central Caribbean,	Among groups	3	0.10	0.01564*
Bahamas,Eastern Caribbean	Among populationswithin groups	15	0.15	0.00293*
	Within populations	1221	99.76	0.05963

AMOVA results showing degrees of freedom (d.f.), variance components (var), percent variation (var%) and F-statistics to test for evidence of regional genetic differentiation among Nassau grouper subpopulations using mitochondrial DNA and microsatellites. (*) denotes statistical significance of *p*<0.05.

**Figure 3:**
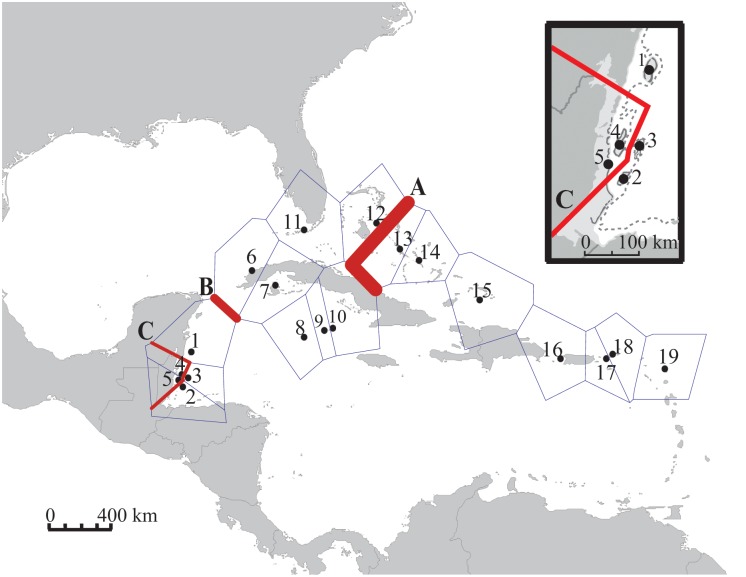
Barriers to larval dispersal in the Caribbean Sea. Genetic barriers between Nassau grouper subpopulations, using Delaunay triangulation and Voronoi tessellation implemented in the software package Barrier. Barriers are ranked in order of impermeability (A through C), with thickness of barrier lines proportional to the frequency with which a given barrier is observed in replicate analyses and indirectly proportional to permeability.

Three barriers to larval dispersal were identified using a computational geometry approach (Fig. 3). The strongest barrier identified (A) separates the eastern Caribbean and western Caribbean between Dog Rocks, N. Exuma and Lee Stocking. The next strongest barrier (B) isolates the Mesoamerican reef from the remainder of the western Caribbean, and the weakest barrier (C) isolates two coastal spawning sites from additional offshore sites in the Mesoamerican reef. Regional clusters defined by the aforementioned barriers (A–C) were genetically distinct from one another (F_ST_ = 0.201, *p* = 0.026). These identified regions roughly confirm patterns seen from *a priori* testing of regional subdivision using AMOVAs.

Results from the simulated annealing approach further support regional genetic subdivision among clusters of subpopulations (Table 5). The mtDNA results indicate maximal variance among groups at *k* = 2, with one group comprised of three localities in Belize (sites 3–5) and another group comprised of all remaining sites (1, 2, 6–19). Microsatellite results indicate maximal variance among groups at *k* = 4. One group is comprised of 2 localities from Belize (sites 4 and 5), a second with the remainder of sampling localities in the Mesoamerican reef and central Caribbean (sites 1–3, 6–11), a third with all localities in the Bahamas (sites 12–14) and a final group with the remaining localities in the eastern Caribbean (sites 15–19).

**Table 5:** AMOVA results from simulated annealing approach.

*mtDNA*	Source of variation	d.f.	var%	*P*-value
(1) Sites 3,4,5 and(2) Sites 1,2, 6–19	Among groups	1	62.9	<0.00001*
	Among populationswithin groups	16	9.84	<0.00001*
	Within populations	377	27.26	0.00196*
(1) Sites 3,4,5, (2) Sites 11–14and (3) Sites 1,2,6–10,15–19	Among groups	2	55.01	<0.00001*
	Among populationswithin groups	15	6.01	<0.00001*
	Within populations	377	38.98	<0.00001*
***Microsatellites***	**Source of variation**	**d.f.**	**var%**	***P*** **-value**
(1) Sites 4,5, (2) Sites 1–3,6–11,(3) Sites 12–14 and (4) 15–19	Among groups	3	0.05	0.00391*
	Among populationswithin groups	15	0.21	0.00880*
	Within populations	1221	99.74	0.03431*

Degrees of freedom (d.f.), variance components (var), percent variation (var%) and *F*-statistics to test for evidence of regional genetic differentiation among Nassau grouper subpopulations using mitochondrial DNA and microsatellites. (*) denotes statistical significance of *p*<0.05.

## Discussion

Despite the challenges of characterizing connectivity among populations in open marine systems, increased understanding of how populations are interconnected across geographic and political landscapes is invaluable for the development of effective management and conservation strategies. In the few genetic studies conducted to date on aggregating fishes in the Caribbean [47], [48], genetic subdivision among subpopulations has not been detected. Lack of population structure was viewed as support for long distance dispersal and extensive mixing among subpopulations of aggregating fishes and offered little support for local management.

We described evidence for strong genetic differentiation among subpopulations of a mass-aggregating marine fish, the Nassau grouper, and detected barriers to larval dispersal in the Caribbean basin. Our findings contribute to the growing body of literature that demonstrates evidence of genetic subdivision among subpopulations of marine species in the Caribbean Sea [49], [50], where there is evidence of limited dispersal in both the larvae and adults of reef fishes. Larval dispersal kernels are not predicted to be greater than 200 km [51] and movement of juveniles and adults are likely to range from 10 km to typically <200 km [52]–[54]. Because these distances are considerably smaller than the average range over which most Caribbean reef fishes occur (approximately 4,000×2,000 km for most species [55]), it is conceivable that there is limited connectivity at the regional scale.

### Regional Patterns of Genetic Differentiation

The Caribbean Sea was once considered a single biogeographic province lacking phylogeographic barriers [56]–[60]. A number of well-known breaks have since been identified through genetic studies, including one between populations east and west of Mona Channel [39], [61], [62] and others isolating the Bahamas [39], [61], [63], [64]. Considering differences in life history among species (i.e. spawning timing, PLD and fecundity), spatial and temporal variation in circulation patterns in the region, as well as variability in sampling schemes between studies, barriers to larval dispersal defined in our work are relatively similar to those indicated for other invertebrate and fish species in the Caribbean basin. We found support for the presence of barriers identified as isolating groups of Nassau grouper subpopulations in the eastern and central Caribbean, Bahamas and Mesoamerican Reef, with no evidence of isolation by distance driving regional patterns. While evidence of variation among sites within regions could be interpreted to mean that the four-region hypothesis is not an exact match to the data, this assumes that populations within regions are panmictic. Observed variance within regional groups could be an indication of some degree of genetic differentiation among spawning aggregations within regions, suggesting the presence of both local and regional population structure.

The strongest barrier (barrier A) detected in our dataset was located in the Bahamas. Much debate exists as to whether the Bahamas represent a distinct genetic enclave. In some genetic studies the Bahamas clustered well with the eastern Caribbean and islands of the Lesser Antilles [65], [66], while a number of hydrodynamic, seascape and population genetic studies provide support for limited dispersal and genetic isolation of Bahamian populations [40], [51], [61]. Pairwise genetic distances, clustering analyses and results from AMOVAs in our study confirm some degree of genetic isolation for Nassau grouper populations in the Bahamas from other sites in the eastern and central Caribbean. Biophysical modeling by Cowen et al. [51] may suggest a potential mechanism driving this divergence. Fishery landings data for Nassau grouper from the Bahamas may also provide evidence of genetic isolation of its stocks. While they have been heavily fished within Bahamian waters and have experienced noticeable decline there [23], the Bahamas represent one of the few remaining areas where substantial landings of Nassau grouper are still obtained in the Caribbean [67]. The extensive continental shelf surrounding the islands provides a large shallow water habitat for Nassau grouper, where there are evidently some remaining aggregations in less accessible (i.e. most distant from fishing centers) locations. The continued presence of these fish in some areas, despite heavy fishing pressures in the region and declining abundances, suggests that Nassau grouper aggregations in the Bahamas are both isolated and potentially self-seeding. Evidence for this notion of potentially self-seeding demes of Nassau grouper is supported by the apparent recovery of Nassau grouper in the Cayman Islands after numerous years of protection [68].

Geographic localities in the central and eastern Caribbean showed varying levels of genetic isolation for Nassau grouper. The central Caribbean has been viewed as a region of mixing, receiving larval inputs from other regions in the Caribbean [39], [41], [51]. There was no evidence of highly divergent subpopulations in this region, thereby confirming findings from oceanographic studies. In contrast, we detected significant evidence of genetic differentiation of eastern Caribbean subpopulations. Genetic isolation of populations in the Lesser Antilles is supported by studies of marine invertebrates, suggesting that the Antilles current may facilitate larval mixing among spawning aggregations off these eastern Caribbean islands [65], [66]. However, we were unable to detect a genetic break between populations on either side of the Mona Channel (off Puerto Rico). An inability to detect this break may be the result of inadequate sampling of subpopulations occupying sites adjacent to the channel.

Of all potential barriers observed in our study, those in the Mesoamerican Reef are the least discussed in the literature [69]–[71]. Pairwise genetic distances, results from the simulated annealing approach and AMOVAs, as well as the large break in the haplotype network, confirm some degree of genetic isolation for Nassau grouper populations in the Mesoamerican Reef. Both barriers B and C, which isolate coastal Belizean aggregation sites and the Mesoamerican Reef as a whole, were more permeable than the barrier observed in the central Bahamas. Permeability of barrier C and weak genetic divergence observed among Mesoamerican reef subpopulations may be explained by fine scale sampling in this region (as few as 40 km between sites). Genetic isolation observed in the Mesoamerican reef is also predicted by oceanographic studies [1], [51]. Currents moving northward along the Central American coastline and cyclonic gyres in the region may suffice to genetically isolate spawning aggregations in the Mesoamerican Reef [72]–[74]. Slower speeds of drogue drifters have been observed in this region as they move northward along the Belizean Mesoamerican reef towards the Yucatan strait [75]; reduced water speeds may facilitate local retention of larvae. Such local larval retention in coastal gyres may be sufficient to explain the genetic distinctness of some spawning aggregations in the Belizean Mesoamerican reef, though further analyses must be conducted to confirm whether local retention is occurring.

Isolation of the aforementioned regional clusters may be best explained by oceanography across the Caribbean. The Caribbean Current represents the strongest flow in the region, moving from the southern Lesser Antilles westward through the Yucatan strait. Mesoscale eddies form within this current and take anywhere from 6 to 10 months to move across the width of the Caribbean, challenging the notion that flow in the region is purely westward [76], [77]. Coupled with knowledge of Nassau grouper’s 35 to 40 day PLD, such eddies may serve to limit Caribbean-wide dispersal of larvae, potentially resulting in the observed regional patterns of genetic subdivision. To directly test this hypothesis, a number of additional methods can be used in future work to investigate whether local retention is the specific mechanism driving patterns of genetic differentiation in Nassau grouper. Results from biophysical modeling studies on ecologically similar mass-aggregating fishes demonstrated that larval retention was maximized in the area surrounding the spawning site [78]. Additionally, DNA parentage analyses studies now allow us to estimate dispersal kernels more directly and to understand dynamics that produce the observed patterns of genetic differentiation [79]–[82]. Work done on an aggregating grouper (coral trout [*Plectropomus areolatus*]) in the Pacific Ocean demonstrated that 50% of larvae remain within 14 km of a given spawning site [83]. In future work, parentage analyses and biophysical modeling parameterized with Nassau grouper larval behavior may provide an excellent means of elucidating mechanisms driving localized patterns of genetic divergence among Nassau grouper subpopulations.

### Alternative Factors Driving Signal of Genetic Differentiation Among Populations

While we believe that large-scale oceanographic patterns best explain regional patterns of genetic differentiation observed, there are other potential biotic and abiotic factors that cannot be discounted. A more than order of magnitude difference between *F*-statistics estimated from the mtDNA versus nuclear markers suggests evidence of sex-biased dispersal or philopatry. While evidence from acoustic tagging studies suggests similar movement and migration patterns in male and female Nassau grouper [84]–[86], there are not enough studies to completely discount this alternative mechanism. Observed genetic differentiation could also be a consequence of the broad time period over which samples were collected. Over a 20-year sampling period, the population structure of a species could potentially shift due to changes in the environment or changes in the intensity of fishing. However, given an average generation time of 9 to 10 years and a maximum life span of 29 years or more [87], we believe it is unlikely that such shifts would be readily reflected in the population structure of Nassau grouper during the sampling period observed. Thus, concordance of barriers with biogeographic patterns observed in the Caribbean for other marine species suggests that regional oceanographic processes likely explain a considerable amount of variation in the datasets.

## Conclusion

We found evidence for strong genetic differentiation among Nassau grouper subpopulations. Our results suggest that the absence of physical barriers to dispersal and potential for long distance dispersal of larvae has not resulted in the genetic homogeneity of Nassau grouper subpopulations throughout the Caribbean Sea. Oceanography likely plays an important role in retaining larvae close to spawning sites at both local and regional spatial scales. Findings warrant additional detailed studies of ocean circulation patterns and dispersal kernels during the spawning period for a more direct investigation of the mechanisms driving genetic divergence among Nassau grouper subpopulations.

Our results nonetheless yield important insights into the vulnerable status of Nassau grouper throughout its geographic range. Spawning aggregations do not withstand heavy and unmanaged fishing, and are not known to reestablish once they are fished out [87]–[89]. If subpopulations represented by spawning aggregations are heavily reliant upon self-recruitment and adults are faithful to specific aggregations, as tagging data suggest, then their persistence, and that of the subpopulations that form them, may rely upon fisheries management and conservation efforts focusing on the maintenance of local genetic diversity and implementing management units at the appropriate spatial scale suggested by genetic data. Regional patterns of genetic differentiation observed may also warrant standardization of fisheries management and conservation initiatives, particularly among countries within genetically isolated regions.

## Supporting Information

File S1 (DOC)
