# Supplementary material for: Population Structure and Phylogeography in Nassau Grouper (Epinephelus striatus), a Mass-Aggregating Marine Fish
Source: PLoS One. 2014 May 15;9(5):e97508. doi: 10.1371/journal.pone.0097508 (PMC4022523; doi:10.1371/journal.pone.0097508)
Supplement: File S1 — (DOC) [file pone.0097508.s001.doc]

**Table S**1. Molecular diversity indices for microsatellite dataset.

| Sampling Sites |  | A113 | D2 | A6 | A10 | A108 | A111 | D8 | A3 | A117 |
| --- | --- | --- | --- | --- | --- | --- | --- | --- | --- | --- |
| 1. Chinchorro Bank | nA | 15 | 13 | 9 | 9 | 7 | 18 | 8 | 14 | 13 |
|  | H­o | 0.917 | 0.833 | 0.792 | 0.875 | 0.750 | 0.958 | 0.625 | 0.750 | 0.875 |
|  | H­e | 0.911 | 0.904 | 0.858 | 0.862 | 0.736 | 0.948 | 0.770 | 0.844 | 0.907 |
| 2. Glover's Reef | nA | 20 | 17 | 10 | 10 | 7 | 27 | 8 | 20 | 16 |
|  | H­o | 0.831 | 0.949 | 0.881 | 0.814 | 0.695 | 0.966 | 0.847 | 0.746 | 0.915 |
|  | H­e | 0.908 | 0.889 | 0.849 | 0.868 | 0.663 | 0.947 | 0.802 | 0.772 | 0.921 |
| 3. Lighthouse Reef | nA | 19 | 12 | 9 | 10 | 8 | 26 | 7 | 15 | 17 |
|  | H­o | 0.938 | 1.000 | 0.938 | 0.781 | 0.719 | 1.000 | 0.906 | 0.781 | 0.969 |
|  | H­e | 0.934 | 0.880 | 0.841 | 0.852 | 0.657 | 0.963 | 0.824 | 0.805 | 0.914 |
| 4. Turneffe Atoll | nA | 17 | 17 | 8 | 7 | 7 | 20 | 10 | 16 | 16 |
|  | H­o | 0.931 | 0.897 | 0.759 | 0.690 | 0.793 | 0.931 | 0.828 | 0.793 | 0.931 |
|  | H­e | 0.930 | 0.901 | 0.844 | 0.852 | 0.740 | 0.953 | 0.818 | 0.852 | 0.913 |
| 5. Caye Glory | nA | 18 | 17 | 14 | 13 | 11 | 22 | 7 | 17 | 16 |
|  | H­o | 0.808 | 0.923 | 0.808 | 0.731 | 0.731 | 0.962 | 0.769 | 0.808 | 0.923 |
|  | H­e | 0.922 | 0.931 | 0.899 | 0.884 | 0.742 | 0.959 | 0.844 | 0.904 | 0.911 |
| 6. Corona San Carlos | nA | 15 | 14 | 7 | 9 | 6 | 21 | 8 | 11 | 12 |
|  | H­o | 0.833 | 0.792 | 0.833 | 0.875 | 0.750 | 0.958 | 0.833 | 0.875 | 0.958 |
|  | H­e | 0.897 | 0.891 | 0.783 | 0.836 | 0.647 | 0.957 | 0.810 | 0.866 | 0.899 |
| 7. Pardon del Medio | nA | 18 | 19 | 10 | 9 | 7 | 29 | 9 | 17 | 17 |
|  | H­o | 0.878 | 0.902 | 0.707 | 0.805 | 0.610 | 0.878 | 0.707 | 0.805 | 0.902 |
|  | H­e | 0.924 | 0.908 | 0.843 | 0.842 | 0.683 | 0.957 | 0.774 | 0.843 | 0.901 |
| 8. Grand Cayman | nA | 11 | 9 | 8 | 7 | 5 | 12 | 7 | 12 | 8 |
|  | H­o | 1.000 | 0.889 | 0.778 | 0.667 | 0.111 | 0.889 | 0.778 | 1.000 | 0.556 |
|  | H­e | 0.935 | 0.902 | 0.902 | 0.810 | 0.752 | 0.948 | 0.876 | 0.954 | 0.791 |
| 9. Little Cayman | nA | 22 | 18 | 12 | 13 | 6 | 24 | 10 | 21 | 20 |
|  | H­o | 0.869 | 0.951 | 0.820 | 0.836 | 0.607 | 0.967 | 0.770 | 0.803 | 0.852 |
|  | H­e | 0.926 | 0.923 | 0.844 | 0.868 | 0.646 | 0.950 | 0.808 | 0.839 | 0.909 |
| 10. Cayman Brac | nA | 16 | 15 | 10 | 11 | 7 | 22 | 10 | 11 | 15 |
|  | H­o | 0.929 | 0.929 | 0.893 | 0.821 | 0.643 | 0.964 | 0.929 | 0.821 | 0.893 |
|  | H­e | 0.932 | 0.919 | 0.836 | 0.851 | 0.641 | 0.959 | 0.846 | 0.849 | 0.881 |
| 11. Florida Keys | nA | 21 | 17 | 10 | 12 | 6 | 24 | 9 | 16 | 17 |
|  | H­o | 0.921 | 0.895 | 0.816 | 0.947 | 0.737 | 0.974 | 0.868 | 0.842 | 0.895 |
|  | H­e | 0.934 | 0.907 | 0.817 | 0.869 | 0.653 | 0.956 | 0.809 | 0.850 | 0.891 |
| 12. Dog Rocks | nA | 14 | 14 | 7 | 8 | 5 | 22 | 9 | 15 | 11 |
|  | H­o | 0.789 | 0.789 | 1.000 | 0.842 | 0.632 | 0.895 | 0.947 | 0.944 | 0.833 |
|  | H­e | 0.910 | 0.902 | 0.834 | 0.765 | 0.637 | 0.964 | 0.841 | 0.921 | 0.854 |
| 13. Lee Stocking | nA | 14 | 14 | 8 | 10 | 5 | 22 | 7 | 14 | 15 |
|  | H­o | 0.739 | 0.913 | 0.609 | 0.913 | 0.565 | 0.957 | 0.696 | 0.913 | 0.913 |
|  | H­e | 0.858 | 0.906 | 0.790 | 0.877 | 0.675 | 0.966 | 0.789 | 0.875 | 0.889 |
| 14. Long Island | nA | 16 | 14 | 10 | 9 | 7 | 22 | 7 | 18 | 17 |
|  | H­o | 0.811 | 0.811 | 0.892 | 0.811 | 0.595 | 0.919 | 0.811 | 0.838 | 0.865 |
|  | H­e | 0.890 | 0.877 | 0.834 | 0.854 | 0.651 | 0.955 | 0.782 | 0.867 | 0.913 |
| 15. South Caicos | nA | 19 | 17 | 10 | 14 | 7 | 25 | 10 | 17 | 18 |
|  | H­o | 0.820 | 0.880 | 0.900 | 0.900 | 0.640 | 0.960 | 0.740 | 0.800 | 0.940 |
|  | H­e | 0.898 | 0.904 | 0.862 | 0.884 | 0.608 | 0.955 | 0.783 | 0.838 | 0.910 |
| 16. Bajo de Sico | nA | 11 | 8 | 7 | 5 | 7 | 13 | 7 | 9 | 9 |
|  | H­o | 0.900 | 1.000 | 1.000 | 1.000 | 0.800 | 1.000 | 0.900 | 0.700 | 1.000 |
|  | H­e | 0.932 | 0.905 | 0.863 | 0.826 | 0.711 | 0.947 | 0.832 | 0.905 | 0.900 |
| 17. Grammanik Bank | nA | 19 | 17 | 10 | 12 | 7 | 24 | 10 | 14 | 17 |
|  | H­o | 0.897 | 0.897 | 0.845 | 0.810 | 0.759 | 0.948 | 0.759 | 0.862 | 0.931 |
|  | H­e | 0.919 | 0.881 | 0.829 | 0.851 | 0.707 | 0.953 | 0.806 | 0.815 | 0.898 |
| 18. N. of St. Thomas | nA | 8 | 9 | 7 | 8 | 4 | 11 | 8 | 8 | 8 |
|  | H­o | 0.500 | 0.875 | 0.875 | 0.875 | 0.250 | 0.875 | 0.750 | 0.875 | 0.875 |
|  | H­e | 0.883 | 0.933 | 0.867 | 0.850 | 0.692 | 0.950 | 0.758 | 0.875 | 0.875 |
| 19. Antigua | nA | 20 | 19 | 10 | 12 | 7 | 24 | 9 | 16 | 17 |
|  | H­o | 0.909 | 0.909 | 0.795 | 0.773 | 0.727 | 0.932 | 0.795 | 0.795 | 0.864 |
|  | H­e | 0.919 | 0.913 | 0.857 | 0.859 | 0.659 | 0.950 | 0.775 | 0.835 | 0.905 |

Number of alleles (nA), observed heterozygosity (H­o­) and expected heterozygosity (H­e) for 9 microsatellite loci.

**Table S2. Pairwise *F***-statistics for concatenated mitochondrial markers and microsatellite loci for Nassau grouper.

|  | 1 | 2 | 3 | 4 | 5 | 6 | 7 | 8 | 9 | 10 | 11 | 12 | 13 | 14 | 15 | 16 | 17 | 18 | 19 |
| --- | --- | --- | --- | --- | --- | --- | --- | --- | --- | --- | --- | --- | --- | --- | --- | --- | --- | --- | --- |
| 1. Chinchorro Bank | --- | 0.004 | 0.002 | 0.000 | -0.002 | 0.001 | -0.003 | 0.004 | -0.001 | 0.001 | -0.001 | 0.002 | -0.001 | 0.004 | 0.006 | 0.001 | 0.005 | 0.014 | 0.007 |
| 2. Glover's Reef | -0.061 | --- | -0.002 | -0.002 | 0.004 | 0.007 | 0.003 | 0.018 | 0.002 | 0.002 | 0.002 | -0.001 | 0.002 | 0.011 | 0.008 | 0.004 | 0.000 | 0.022 | 0.003 |
| 3. Lighthouse Reef | 0.378 | 0.344 | --- | -0.001 | 0.000 | 0.004 | 0.000 | 0.007 | 0.000 | -0.004 | -0.002 | -0.002 | -0.001 | 0.006 | 0.007 | 0.002 | 0.001 | 0.016 | -0.001 |
| 4. Turneffe Atoll | 0.404 | 0.356 | **0.180** | --- | 0.001 | 0.004 | 0.002 | 0.012 | 0.001 | 0.000 | 0.000 | -0.004 | 0.001 | 0.004 | 0.005 | 0.003 | 0.002 | 0.001 | -0.003 |
| 5. Caye Glory | 0.537 | 0.428 | 0.086 | 0.028 | --- | 0.005 | 0.002 | 0.005 | 0.000 | -0.001 | 0.003 | 0.000 | 0.006 | 0.001 | 0.005 | 0.001 | 0.004 | 0.010 | 0.005 |
| 6. Corona San Carlos | **0.407** | 0.302 | 0.937 | **0.936** | **0.800** | --- | 0.006 | 0.018 | 0.004 | 0.000 | -0.001 | 0.000 | 0.005 | 0.006 | 0.005 | 0.009 | 0.001 | 0.022 | 0.004 |
| 7. Pardon del Medio | 0.161 | 0.146 | **0.760** | **0.766** | **0.933** | 0.021 | --- | 0.003 | -0.003 | -0.001 | -0.002 | -0.002 | 0.000 | -0.001 | 0.007 | 0.003 | 0.001 | 0.014 | 0.001 |
| 8. Grand Cayman | 0.301 | 0.250 | 0.922 | 0.922 | **0.925** | 0.000 | -0.021 | --- | 0.010 | 0.005 | 0.068 | 0.011 | 0.015 | -0.003 | 0.020 | 0.008 | 0.014 | 0.026 | 0.013 |
| 9. Little Cayman | **0.574** | **0.477** | **0.915** | **0.915** | **0.916** | 0.083 | -0.021 | -0.028 | --- | -0.002 | -0.002 | -0.002 | -0.001 | 0.000 | 0.005 | 0.003 | 0.000 | 0.014 | -0.001 |
| 10. Cayman Brac | **0.507** | **0.376** | **0.937** | **0.937** | **0.935** | 0.054 | -0.029 | -0.003 | -0.014 | --- | -0.006 | -0.002 | 0.002 | -0.002 | 0.007 | 0.006 | 0.003 | 0.019 | -0.002 |
| 11. Florida Keys | -0.059 | -0.026 | 0.416 | 0.425 | **0.481** | 0.217 | 0.081 | 0.173 | **0.357** | **0.272** | --- | -0.006 | -0.001 | -0.001 | 0.004 | 0.004 | -0.002 | 0.016 | -0.002 |
| 12. Dog Rocks | -0.174 | -0.152 | 0.315 | 0.355 | **0.519** | 0.538 | 0.228 | 0.417 | 0.675 | 0.636 | -0.104 | --- | 0.002 | 0.005 | 0.004 | -0.005 | -0.004 | 0.008 | -0.001 |
| 13. Lee Stocking | 0.201 | 0.192 | 0.965 | 0.960 | 0.944 | 0.038 | -0.045 | -0.089 | -0.126 | 0.525 | 0.114 | 0.307 | --- | 0.010 | 0.005 | 0.004 | 0.001 | 0.018 | 0.002 |
| 14. Long Island | 0.017 | 0.062 | 0.583 | 0.595 | **0.646** | 0.096 | -0.014 | 0.057 | **0.196** | 0.138 | 0.019 | 0.057 | -0.008 | --- | 0.006 | 0.006 | 0.007 | 0.018 | 0.001 |
| 15. South Caicos | 0.027 | 0.052 | 0.573 | 0.580 | **0.623** | 0.197 | -0.114 | 0.054 | **0.186** | 0.133 | 0.010 | 0.041 | 0.001 | -0.024 | --- | 0.007 | 0.006 | 0.018 | 0.006 |
| 16. Bajo de Sico | -0.050 | -0.002 | 0.187 | 0.205 | **0.306** | **0.537** | **0.340** | **0.456** | **0.699** | **0.626** | 0.057 | -0.143 | 0.388 | 0.187 | 0.195 | --- | 0.005 | 0.018 | 0.007 |
| 17. Grammanik Bank | **0.602** | **0.494** | **0.929** | **0.929** | **0.929** | -0.122 | 0.095 | -0.020 | -0.003 | -0.006 | **0.368** | 0.707 | -0.030 | 0.209 | **0.198** | **0.720** | --- | 0.018 | -0.002 |
| 18. North of St. Thomas | N/A | N/A | N/A | N/A | N/A | N/A | N/A | N/A | N/A | N/A | N/A | N/A | N/A | N/A | N/A | N/A | N/A | --- | 0.007 |
| 19. Antigua | 0.015 | 0.058 | 0.109 | 0.091 | **0.167** | **0.476** | **0.353** | **0.434** | **0.629** | **0.537** | **0.118** | -0.080 | 0.392 | 0.244 | 0.249 | -0.053 | 0.641 | N/A | --- |

Pairwise ST ­­values for mitochondrial markers are below diagonal and pairwise F­ST­ values for microsatellites are above diagonal. Bolded values denote statistical significance of *p* < 0.00029 (mtDNA) and *p* < 0.00027 (microsatellites), using Bonferroni correction for multiple tests. N/A indicates pairwise comparisons that could not be generated due to poor amplification of mtDNA.

**Table S3. Pairwise *F*-statistics for 4,234 SNP loci for Nassau grouper.**

|  | 2 | 9 | 16 | 19 |
| --- | --- | --- | --- | --- |
| 2. Glover's Reef | --- |  |  |  |
| 9. Little Cayman | **0.003** | --- |  |  |
| 14. Long Island | **0.004** | **0.001** | --- |  |
| 19. Antigua | **0.006** | 0.001 | **0.003** | --- |

Pairwise FST values. Bolded values denote statistical significance of *p* < 0.00512 (Bonferroni correction for multiple tests).
